# New integrated weaning indices from mechanical ventilation: A derivation-validation observational multicenter study

**DOI:** 10.3389/fmed.2022.830974

**Published:** 2022-07-22

**Authors:** Amir Vahedian-Azimi, Keivan Gohari-Moghadam, Farshid Rahimi-Bashar, Abbas Samim, Masoum Khoshfetrat, Seyyede Momeneh Mohammadi, Leonardo Cordeiro de Souza, Ata Mahmoodpoor

**Affiliations:** ^1^Trauma Research Center, Nursing Faculty, Baqiyatallah University of Medical Sciences, Tehran, Iran; ^2^Medical ICU and Pulmonary Unit, Shariati Hospital, Tehran University of Medical Sciences, Tehran, Iran; ^3^Anesthesia and Critical Care Department, Hamadan University of Medical Sciences, Hamadan, Iran; ^4^Chemical Injuries Research Center, Systems Biology and Poisonings Institute, Baqiyatallah University of Medical Sciences, Tehran, Iran; ^5^Department of Anesthesiology and Critical Care, Khatamolanbia Hospital, Zahedan University of Medical Sciences, Zahedan, Iran; ^6^Department of Anatomical Sciences, School of Medicine, Zanjan University of Medical Sciences, Zanjan, Iran; ^7^Physical Therapy College, Universidade Estáciode Sá, Rio de Janeiro, Brazil; ^8^Evidence Based Medicine Research Center, Tabriz University of Medical Sciences, Tabriz, Iran

**Keywords:** mechanical ventilation, weaning indices, cut-off values, likelihood ratio, receiver-operating characteristic curve

## Abstract

**Background:**

To develop ten new integrated weaning indices that can predict the weaning outcome better than the traditional indices.

**Methods:**

This retrospective-prospective derivation-validation observational multicenter clinical trial (Clinical Trial.Gov, NCT 01779297), was conducted on 1,175 adult patients admitted at 9 academic affiliated intensive care units (ICUs; 4 surgical and 5 medical), from Jan 2013 to Dec 2018. All patients, intubated and mechanically ventilated for at least 24 h and ready for weaning were enrolled. The study had two phases: at first, the threshold values of each index that best discriminate between a successful and an unsuccessful weaning outcome was determined among 208 patients in the derivation group. In the second phase, the predictive performance of these values was prospectively tested in 967 patients in the validation group. In the prospective-validation set we used Bayes’ theorem to assess the probability of each test in predicting weaning.

**Results:**

In the prospective validation group, sensitivity, specificity, diagnostic accuracy, positive and negative predictive values, and finally area under the receiver operator characteristic curves and standard errors for each index (ten formulae) were calculated. Statistical values of ten formulae for aforesaid variables were higher than 87% (0.87–0.99).

**Conclusion:**

The new indices can be used for hospitalized patients in intensive care settings for accurate prediction of the weaning outcome.

## Introduction

Mechanical ventilation (MV) is a life-supporting modality that is used in many critically ill patients and it aims to support ventilation, optimize oxygenation, and protect the airway (1). Weaning from MV is a particularly important issue because early and late extubation will put pressure on the patient’s health, increase the risk of infection and length of stay in hospital (2). As many as 20% of mechanically ventilated patients may fail their first attempt to disconnect from mechanical ventilation. Weaning can account for more than 40–50% of the total duration of MV (3–5). Long-term MV is associated with many complications such as ventilator-associated pneumonia (VAP), ventilator-induced lung injury, airway injury, ventilator-induced diaphragmatic dysfunction, barotraumas, and prolonged immobility sequelae (6–8). According to the Sixth International Consensus Conference on Intensive Care Medicine (9), patients who meet the following criteria should be considered as potentially ready for liberation from the ventilator, a frequency to tidal volume ratio (f/Vt) less than 105 breaths/min/L, respiratory rate (f) of 35 breaths/min or less, maximal inspiratory pressure (MIP) of equal to or more negative than -20/-25 cm/H_2_0, spontaneous tidal volume (Vt) more than 5 mL/kg, vital capacity more than 10 mL/kg, and arterial oxygen saturation (SaO_2_) > 90% with a fraction of inspired oxygen (FiO_2_) of 0.4 or less (or partial pressure of arterial oxygen (PaO_2_)/FiO_2_ ratio of 150 mmHg or more) (10–13).

Challenges to weaning indices development include differences in patient populations and pathophysiologic conditions, variable techniques of measurements, and lack of objective criteria to define the weaning outcomes (14–16). Discontinuation of mechanical ventilation, spontaneous breathing trial (SBT), and extubation are carried out under the attending clinician’s evaluation, arterial blood gas analysis, and observation of the patient’s clinical condition. For example, the majority of clinicians use the measurement of F/Vt ratio after sustained low or absent pressure support and Positive End-Expiratory Pressure (PEEP), the so-called rapid shallow breathing index (RSBI) for both restrictive and obstructive patients (17, 18). In addition, diagnostic tools to evaluate respiratory muscle function could be helpful to guide the start of the weaning process (19, 20). Despite RSBI has been validated in so many studies and found to have an excellent accuracy, but the RSBI value is questionable in medical critically ill patients and during sedation with some drugs like propofol. In addition, single measurement of RSBI can be misleading and repeated measurement of RSBI increase its accuracy in weaning failure and an increasing RSBI was noted in weaning failures. Also, RSBI that measured during spontaneous breathing trial with spirometry has different accuracy from RSBI that measured during pressure support ventilation. So, it can be suggested that RSBI may not have adequate accuracy to be used routinely in the weaning process and the quest to obtain an accurate way to predict success when weaning a patient from mechanical ventilation continues (21–24).

This study was conducted to introduce and compare ten weaning indices in predicting successful weaning from MV. In the search for an index with better predictive power for MV liberation we considered the combination of parameters. The idea of formulation of the new integrative indices that evaluate different pathophysiology and weaning failure mechanisms can improve the predictive power of simple weaning indices.

## Materials and methods

### Study design

This was a retrospective-prospective derivation-validation observational multicenter clinical trial conducted in 9 academic affiliated intensive care units (ICUs; 4 surgical and 5 medical) from Jan 2013 to Dec 2018 develop ten new integrated weaning indices that can predict the weaning outcome better than the traditional indices. All parts of study were reviewed according to the Strengthening the Reporting of Observational Studies in Epidemiology for Respondent-Driven Sampling Studies: “STROBE-RDS” Statement. The study protocol was approved by the investigational review boards at each of all participating centers in Iran, and written informed consent from each patient or their legal representative was obtained before any study procedures.

### Participants

This study enrolled all adult patients admitted to 9 academic affiliated intensive care units (ICUs; 4 surgical and 5 medical), from Jan 2013 to Dec 2018. Patients were eligible for study participation if: (a) age ≥ 18 years, (b) admitted to the ICU, (c) endotracheal intubated and on mechanical ventilation for ≥ 24 h, (d) full-code status, and if (e) informed consent was provided by the patient, legal guardian, or healthcare surrogate (before ventilator weaning). Patients were excluded for: (a) declining consent, (b) death without ventilator weaning, (c) cardiopulmonary arrest on the ventilator, (d) permanent ventilator dependence, (e) tracheostomy placement for long-term weaning, (f) self-extubation, (g) aspiration during the wean, (h) copious secretions and mucus plugging precluding wean, and (i) incomplete data.

### Setting of study

All patients were intubated with tracheal tubes mechanically ventilated for at least 24 h and ready for weaning. The ventilators used were the Evita XL and Evita 4 edition ventilators (Draeger, Lubeck, Germany). All intubated patients in this study were divided into two groups, derivation, and validation groups. The study had two phases: at first, retrospectively the threshold values of each index that best discriminate between a successful and an unsuccessful weaning outcome was determined among the derivation group. In the second phase, prospectively the predictive performance of these values was tested in the validation group.

### New integrated weaning indices

We developed ten indices by combining different respiratory variables and different simple weaning indices. For the determination of the best performance of these variables, we invited three-expert of panels. The members of these panels included pulmonary diseases consultants, specialists in anesthesiology, and intensivists from different country regions. Sections one and two were held by posting indices on the internet looking for their predictive values among different studies and after all, opinions were collected for section three, we invited them to participate in the 120-min focus group. Finally, ten new indices emerged as follow:

Index 1 = (PPR)/(RSBI × F_i_O_2_)
Index 2 = (PPR)/(RSBI × F_i_O_2_ × P0.1)
Index 3 = (PPR × NIF)/(RSBI × F_i_O_2_)
Index 4 = (PPR × NIF)/(RSBI × F_i_O_2_ × P0.1)
Index 5 = (NIF)/(P0.1)
Index 6 = (S_a_O_2_)/[(P (A-a) O_2_ × RSBI × F_i_O_2_]
Index 7 = (S_a_O_2_)/[(P (A-a) O_2_ × RSBI × F_i_O_2_ × P0.1]
Index 8 = (S_a_O_2_ × NIF)/[(P (A-a) O_2_ × RSBI × F_i_O_2_]
Index 9 = (S_a_O_2_ × NIF)/[(P (A-a) O_2_ × RSBI × F_i_O_2_ × P0.1]
Index 10 = (S_a_O_2_)/[(P (A-a) O_2_ × P0.1]
(N.B: Where PPR = PaO2: PAO2 ratio)

For computing new indices, a calculator is designed to calculate new indices. Our recommended indices use four essential parameters that lend themselves to easy measurement and are independent of the patient’s cooperation. The scores, in a single equation, the respiratory system dynamics, the respiratory drive, the oxygenation/ventilation, and the respiratory pattern, through NIF, P0.01, PPR-P(A-a) O2, SaO2 and RSBI ratio respectively. The operation with this calculator was so simple because seven variables should enter into the calculator for computing ten formulae (FiO2, PaO2, SaO2, PaCO2, RSBI, P0.1, and NIF). Baseline demographics, initial diagnosis, and pre-extubation clinical data are collected for each patient.

### Weaning procedure

Liberation of MV was attempted when the primary physician judged that the patient was ready for a spontaneous breathing trial (SBT), according to the following criteria: competent airway, good cough reflex, absence of sedation, or excessive tracheal secretions, and hemodynamic stability. Sedation was discontinued before the evaluation of weaning. Patients who met these criteria were initially placed on SBT (continuous positive airway pressure of 5 mmHg, FiO2 ≤ 0.4) for 3 min to obtain weaning variables at the end of SBT. If Oxygen saturation ≥ 92% on pulse oximetry with FiO2 ≤ 0.4 and RSBI < 105 breaths/min/L, patients were continued on SBT for 30 min during which clinical variables and ventilator variables were monitored closely for signs of respiratory distress (respiratory rate > 30 breaths/min, SaO2 < 90%, heart rate > 140 breaths/min, or a sustained increase or decrease of heart rate of > 20%, blood pressure > 200 mm Hg or < 80 mm Hg, and agitation, diaphoresis, or anxiety). At the end of the SBT, the RSBI was measured again, arterial blood gas (ABG) was obtained, and the predetermined values are calculated and measured. The decision to reinstitute MV was made based on airway competence (cough, sputum production, neurologic status, level of consciousness, and MIP) (25). Patients who remain extubated for 24 h are classified as successful extubation without extra helping including more oxygenation, reintubation, or using Non-invasive MV. Weaning failure considered if patients need more support during 24 h after extubation including more oxygenation, reintubation, or using Non-invasive MV.

### Statistical analyses

Data were presented using mean ± standard division (SD) or medians (inter-quartile range, IQR), for continuous variables and frequencies with percentages (%) for categorical characteristics. The whole data was split into two subsamples: derivation data and validation data. To compare the differences in terms of demographic characteristics, clinical data, incidence of successful and failure weaning between derivation and validation groups *t*-test and Chi-square test were used for distributed continuous and categorical variables, respectively. Association with the success weaning was tested by univariate and multivariate logistic regression analysis using the “Enter” method as the independent variable of primary interest. In models, the odds ratio (OR) and their 95% confidence interval (CI) were reported as the effect size of the association. Based on the coefficients derived from the model in the derivation dataset, the scores were computed for the validation data set. The model validation was assessed in the validation data set utilizing diagnostic accuracy measures and their 95% CI by receiver operating characteristic (ROC) curves analysis and calculate their area under the curves (AUC), alongside sensitivity (SN), specificity (SP), positive predictive value (PPV), negative predictive value (NPV), positive likelihood ratio (LR+), negative likelihood ratio (LR−), accuracy and Youden score to find appropriate cut-offs. In the tables with zero counts, likelihood ratios were estimated using the substitution formula and 0.5 was added to all cell frequencies before calculation. According to general guide for the discriminative power of a test based on ROC, AUC between (0.9–1.0), (0.8–0.9), (0.7–0.8), and (0.6–0.7) was considered as excellent, good, fair, and poor, respectively. In addition, the AUCs was compared by DeLong test. All analyses were conducted using STATA software ver.13 (Stata Corp., College Station, TX, United States), SPSS software (ver.21) (SPSS Inc., Chicago, IL, United States) and MedCalc for ROC analysis. In all analyses, *P*-values less than 0.05 were considered significant.

## Results

### Clinical characteristics and outcomes of the study population

**FIGURE 1:**
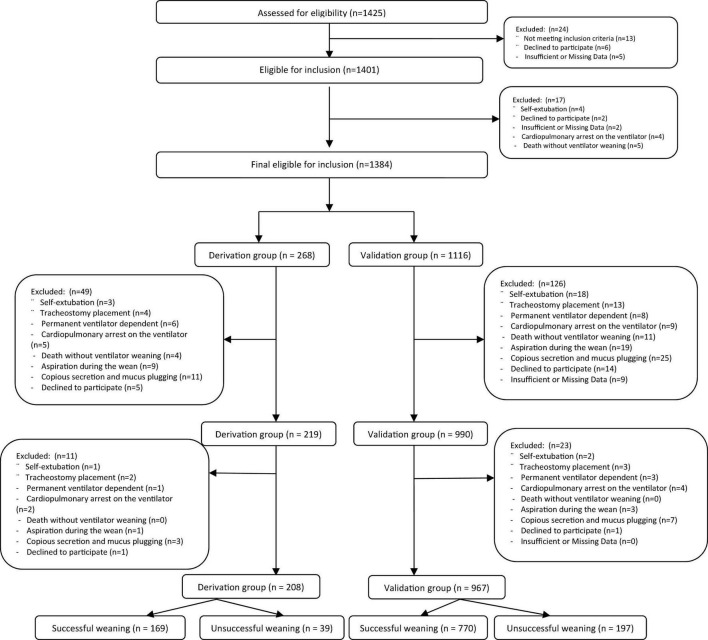
The study population flowchart.

From Jan 2013 to Dec 2018, a total of 1,175 patients were screened at 9 academic affiliated intensive care units (ICUs; 4 surgical and 5 medical) in Iran. All 1,175 intubated patients in this study were divided into two groups, derivation (*n* = 208) and validation (*n* = 967) groups (Figure 1). Demographic data, clinical characteristics, incidence of successful and failure weaning in both derivation, and validation groups and total population are presented in Table 1. The mean ± SD of all participant ages was 58.36 ± 7.94 years. There were 523 (44.5%) male patients and 652 (55.5%) female patients. According to the results, the mean age of validation group was significantly higher than the derivation group (58.82 ± 7.57 vs. 56.19 ± 9.16, *P* < 0.001). However, no significant difference was observed between groups according to gender (*P* = 0.929). Patients were admitted to ICUs for various reasons including cancer, acute respiratory distress syndrome (ARDS), chronic obstructive pulmonary disease (COPD), multiple traumas, abdominal surgery, pneumonia, and sepsis. However, there was no significant difference between groups according to cause of ICU admission (*P* = 0.838). The most common cause of ICU admission for both groups was COPD (validation: 23.7% vs. derivation: 21.2%, *P* = 0.745). Illness severity as measured by the Acute Physiology and Chronic Health Evaluation (APACHE) II, Sequential Organ Failure Assessment (SOFA), and Simplified Acute Physiology Score (SAPS) scores, which the mean ± SD scores of all participants was 26.43 ± 4.83, 14.90 ± 3.86, and 49.11 ± 4.64, respectively. No significant differences were observed between groups according to APACHE II (26.35 ± 5.63 vs. 26.44 ± 4.64, *P* = 804), SOFA (14.20 ± 3.01 vs. 15.27 ± 3.92, *P* = 0.052), and SPAS (48.56 ± 49.22, *P* = 0.060) scores. The prevalence of extubation failure in all patients was 20.1% and no significant difference was observed between groups in terms of weaning rate (18.8% vs. 20.4%, *P* = 0.569). However, the mean ± SD of ICU (*P* < 0.001) and hospital (P-0.034) length of stay (LOS) were significantly higher in the validation group than that the derivation group. Pulmonary-related variables, gas exchange-related variables, conventional weaning indices, and the mean ± SD of 10 new integrated weaning indices were observed in Table 1 can be seen in detail in both derivation and validation data sets groups.

**TABLE 1:** Demographic data, clinical characteristics, incidence of successful and failure weaning in both derivation and validation groups and total population.

Variables	Total Population (*n* = 1,175)	Derivation dataset (*n* = 208)	Validation dataset (*n* = 967)	*P*-value
**Gender** Male, N (%) Female, N (%)	523 (44.5) 652 (55.5)	92 (44.2) 116 (55.8)	431 (44.6) 536 (55.4)	0.929
**The cause of admission** Cancer, N (%) ARDS, N (%) COPD, N (%) Multiple traumas, N (%) Abdominal surgery, N (%) Pneumonia, N (%) Sepsis, N (%)	97 (8.3) 216 (18.4) 273 (23.2) 223 (19) 193 (16.4) 119 (10.1) 54 (4.6)	14 (6.7) 39 (18.8) 44 (21.2) 38 (18.3) 38 (18.3) 23 (5.8) 12 (5.8)	83 (8.6) 177 (18.3) 229 (23.7) 185 (19.1) 155 (16) 96 (9.9) 42 (4.3)	0.838
**Weaning rate** Successful, N (%) Unsuccessful, N (%)	939 (79.9) 236 (20.1)	169 (81.3) 39 (18.8)	770 (79.6) 197 (20.4)	0.569
Age, Year, Mean (SD)	58.36 (7.94)	56.19 (9.16)	58.82 (7.57)	**<0.001**
**ICU-related variables** ICU LOS, Day, Mean (SD) Hospital LOS, Day, Mean (SD) APACHE II, Mean (SD) SOFA, Mean (SD) SAPS, Mean (SD) Hemoglobin, g/dl, Mean (SD)	23.73 (7.96) 13.56 (5.06) 26.43 (4.83) 14.90 (3.86) 49.11 (4.64) 11.13 (1.54)	19.71 (8.17) 12.89 (5.60) 26.35 (5.63) 14.20 (3.01) 48.56 (4.90) 10.98 (1.50)	24.59 (7.64) 13.71 (4.93) 26.44 (4.64) 15.27 (3.92) 49.22 (4.57) 11.17 (1.55)	**<0.001** **0.034** 0.804 0.052 0.060 0.118
**Pulmonary-related variables** C_Dynamic_, ml/cmH_2_O, Mean (SD) C_Static_, ml/cmH_2_O, Mean (SD) VE, l/min, Mean (SD) VT, ml/min, Mean (SD) RR, breath/min, Mean (SD)	23.39 (3.63) 36.16 (6.42) 7.84 (1.43) 391.31 (40.69) 20.01 (2.82)	23.10 (3.60) 36.94 (5.75) 8.61 (1.75) 422.31 (39.37) 20.34 (3.36)	23.46 (3.64) 35.99 (6.54) 7.67 (1.30) 384.64 (37.79) 19.94 (2.69)	0.192 0.053 **<0.001** **<0.001** 0.063
**Gas exchange-related variables** PaO_2_, mmHg, Mean (SD) SaO_2_,%, Mean (SD) PaCO_2_, mmHg, Mean (SD) FiO_2_,%, Mean (SD) P_ALV_O_2_, mmHg, Mean (SD) PaO_2_/P_ALV_O_2_, mmHg, Mean (SD) P(ALV-a) O_2_, mmHg, Mean (SD)	88.76 (5.30) 88.89 (1.47) 43.92 (2.73) 35.27 (2.27) 159.86 (14.30) 0.56 (0.06) 71.10 (15.10)	84.31 (3.60) 89.03 (1.56) 43.57 (2.88) 35.39 (2.28) 161.04 (1453) 0.53 (0.05) 76.73 (14.57)	89.72 (5.19) 88.87 (1.45) 44 (2.69) 35.24 (2.26) 159.61 (14.24) 0.57 (0.06) 69.89 (14.95)	**<0.001** 0.144 **0.035** 0.404 0.191 <0.001 <0.001
**Conventional weaning indices** RSBI, breath/l/min, Mean (SD) NIF, cmH_2_O, Mean (SD) P.01, milli/second, Mean (SD)	90.39 (10.62) 23.72 (2.97) 5.72 (1.91)	92.74 (10.89) 24.63 (2.90) 6.49 (2.34)	89.89 (10.50) 23.53 (2.96) 5.55 (1.76)	**<0.001** **<0.001** **<0.001**
**New integrated weaning indices** 1. (PPR/RSBI*FiO_2_), Mean (SD) 2. (PPR/RSBI*FiO_2_*P.01), Mean (SD) 3. (PPR*NIF/RSBI*FiO_2_), Mean (SD) 4. (PPR*NIF/RSBI*FiO_2_*P.01), Mean (SD) 5. (NIF/P.01), Mean (SD) 6. (SaO_2_/P(ALV-a) O_2_*RSBI*FiO_2_), Mean (SD) 7. (SaO_2_/P(ALV-a) O_2_*RSBI*FiO_2_*P.01), Mean (SD) 8. (SaO_2_*NIF/P(ALV-a) O_2_*RSBI*FiO_2_), Mean (SD) 9. (SaO_2_*NIF/P(ALV-a) O_2_*RSBI*FiO_2_*P.01), Mean (SD) 10. (SaO_2_/P(ALV-a) O_2_*P.01), Mean (SD)	180.28 (40.34) 350.64 (133.86) 426.29 (104.02) 821.33 (305.06) 454.74 (132.84) 430.59 (166.75) 838.89 (424.58) 1016.78 (402.91) 196.23 (96.93) 256.81 (105.24)	164.80 (33.66) 290.46 (117.33) 405.25 (93.04) 712.75 (289.14) 435.52 (162.32) 380.26 (120.34) 669.65 (315.43) 933.82 (306.88) 164.10 (77.03) 214.01 (92.11)	183.62 (40.89) 363.59 (133.71) 430.82 (105.73) 844.68 (303.48) 458.87 (125.32) 441.42 (173.28) 875.29 (436.20) 1034.63 (418.70) 203.14 (99.39) 266.01 (105.65)	**<0.001** **<0.001** **<0.001** **<0.001** **0.021** **<0.001** **<0.001** **<0.001** **<0.001** **<0.001**

P-values of 0.05 are shown in bold and are considered significant.

### Findings of logistic regression analysis

Univariate and multivariate Binary logistic regression analysis to determine the effect of demographic characteristics and clinical data on outcome of weaning are presented in Table 2. However, according to the results, we not found any statistical significance between the variables and the weaning outcome.

**TABLE 2:** Univariate and multivariate logistic regression analysis to determine the effect of demographic characteristics and clinical data on weaning outcome.

Variables	Univariate		Multivariate	
	OR (95% CI)	*P*-value	OR (95% CI)	*P*-value
Age	1.002 (0.984–1.02)	0.817	1.003 (0.985–1.022)	0.749
Gender (Female vs. male)	0.957 (0.718–1.276)	0.764	0.96 (0.718–1.282)	0.78
APACHE II	1.003 (0.973–1.033)	0.861	1.003 (0.973–1.033)	0.867
SOFA	0.991 (0.955–1.028)	0.627	0.994 (0.957–1.033)	0.769
SAPS	1.006 (0.975–1.037)	0.705	1.008 (0.977–1.04)	0.601
ICU LOS	0.986 (0.968–1.004)	0.13	0.986 (0.968–1.005)	0.154
Hospital LOS	0.979 (0.952–1.006)	0.132	0.98 (0.953–1.008)	0.152
Cause of ICU admission	1.021 (0.934–1.116)	0.648	1.024 (0.937–1.121)	0.597
Groups (derivation vs. validation)	1.109 (0.757–1.624)	0.596	1.02 (0.68–1.53)	0.925

OR, odds ratio; CI, confidence interval.

### Results on derivation sample

**FIGURE 2:**
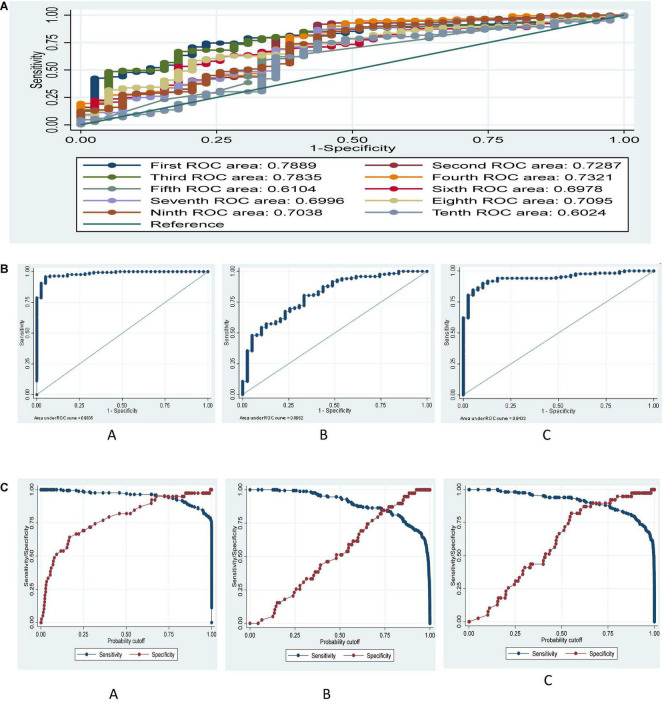
**(A)** ROC curve for ten formulae based on derivation data set. Ho: area (First) = area (Second) = area (Third) = area (Fourth) = area (Fifth) = area (Sixth) = area (Seventh) = area (Eighth) = area (Ninth) = area (Tenth), chi^2^ (9) = 205.6, *P* < 0.001, **(B)** ROC curve for **(A)** ten **(A)**, first five **(B)**, and second five **(C)** new integrated weaning indices after multiple logistic regression based on the derivation data set. **(C)** ROC curve for sensitivity and specificity tradeoff for ten **(A)**, first five **(B)**, and 2nd five **(C)** new integrated weaning indices after multiple logistic regression based on derivation data set.

Comparison of the AUCs of ten new integrated weaning indices for predicting successful weaning are presented in Figure 2A. Best performing predictive value for successful weaning were related to the first and third formulas with (AUC: 0.788, 95% CI: 0.727–0.842, *P* < 0.001), and (AUC: 0.783, 95% CI: 0.721–0.837, *P* < 0.001), respectively. However, no significant difference was observed between AUCs of first and third formula (0.788 vs. 0.783, *P* = 0.779). Poor predictive value for successful weaning was related to the fifth formula with (AUC: 0.610, 95% CI: 0.541–0.677, *P* = 0.035). Predictive value of tenth formula was not significant (AUC: 0.602, 95% CI: 0.532–0.669, *P* = 0.067). The results according to DeLong test indicated a significant difference of AUCs among fifth and tenth formulas with the others (*P* < 0.05). The ROC area for ten, first 5, and second five new integrated weaning indices are presented in Figure 2B. The results indicated AUC of about 0.98, 0.81, and 0.94 respectively, for ten, first five, and second five new integrated weaning indices simultaneously on predicting successful weaning after fitting a multiple logistic regression in derivation dataset.

### The cumulative effect of all new integrated weaning indices in derivation dataset

**FIGURE 3:**
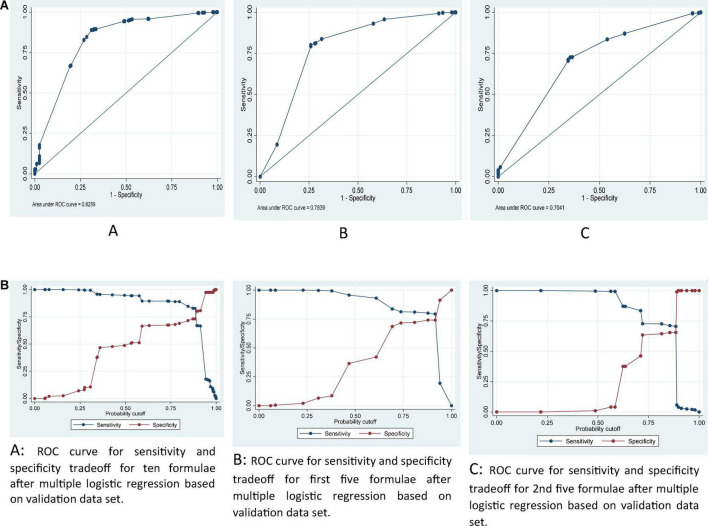
**(A)** ROC curve for ten **(A)**, first five **(B)** and 2nd five **(C)** new integrated weaning indices after multiple logistic regression based on validation data set. **(B)** ROC curve for sensitivity and specificity tradeoff for ten **(A)**, first five **(B)**, and 2nd five **(C)** new integrated weaning indices after multiple logistic regression based on validation data set.

To have a cumulative effect of all indices, we conducted a logistic regression, and then using the probability of successful weaning in this model, we computed the diagnostic indices. Diagnostic indices for this model considering 0.5 and optimal cutoffs for predicted probability is shown in Table 3. Diagnostic indices in derivation dataset indicated that the model by new integrated weaning indices had higher accuracy, SN, SP, LR +, PPV, and NPV and lower values of LR− in both 0.5 and optimal cutoff values as compared to two other sets of formulae. Additionally, model by second 5 formulae had higher accuracy, SN, SP, LR +, PPV, and NPV and lower values of LR- in both 0.5 and optimal cutoff values as compared to the model by first five formulae. The optimal cutoff values were estimated based on sensitivity and specificity tradeoff in Figure 3. The Figure 2C showed a tradeoff of sensitivity and specificity in the cutoff of around 0.7, 0.8, and 0.7 for predicted probability in the model by ten, first five and 2nd five new integrated weaning indices, respectively, based on the multivariate logistic regression.

**TABLE 3:** Diagnostic indices after multiple logistic regression model based on derivation data set.

Model	SN (95% CI)	SP (95% CI)	LR + (95% CI)	LR- (95% CI)	PPV (95% CI)	NPV (95% CI)	Youden Index	Accuracy
LR on 10 formulae, cut point = 0.5	97 (93–99)	82 (67–93)	5.4 (2.8–10.6)	0.04 (0.02–0.09)	96 (92–98)	87 (71–96)	0.79	94.2
LR on 10 formulae, Optimal cut point = 0.7	95 (91–98)	95 (83–99)	18.6 (4.8–71.7)	0.05 (0.03–0.1)	99 (96–100)	82 (68–92)	0.90	95.2
LR on 1–5 formulae, cut point = 0.5	96 (92–98)	31 (17–48)	1.4 (1.1–1.7)	0.14 (0.06–0.30)	86 (80–90)	63 (38–84)	0.27	83.7
LR on 1–5 formulae, Optimal cut point = 0.8	78 (71–84)	67 (50–81)	2.3 (1.5–3.7)	0.34 (0.24–0.48)	91 (85–95)	41 (29–54)	0.45	75.5
LR on 6–10 formulae, cut point = 0.5	94 (89–97)	69 (52–83)	3.1 (1.9–4.9)	0.09 (0.05–0.16)	93 (88–96)	73 (56–86)	0.63	89.4
LR on 6–10 formulae, Optimal cut point = 0.7	89 (83–93)	90 (76–97)	8.7 (3.4–21.9)	0.13 (0.08–0.19)	97 (94–99)	65 (51–77)	0.79	88.9

LR10, Logistic regression on 10 formulae; LR 1_5, Logistic regression on first 5 formulae; LR 6_10, Logistic regression on second 5 formulae; Cut, cut point; Optimal cut point, optimal cut point based on sensitivity and specificity in the ROC curve after logistic; CI, Confidence interval; SN, Sensitivity; SP, Specificity; LR +, Positive Likelihood Ratio; LR-, Negative Likelihood Ratio; PPV, Positive Predictive value; NPV, Negative Predictive value.

### Results on the validation sample

Good performing predictive value for successful weaning in the validation group were related to the first and third formulas with (AUC: 0.846, 95% CI: 0.822–0.869, *P* < 0.001), and (AUC: 0.828, 95% CI: 0.803–0.851, *P* < 0.001), respectively. Fair performing predictive were related to the sixth and eight formulas with (AUC: 0.737, 95% CI: 0.708–0.764, *P* < 0.001), and (AUC: 0.730, 95% CI: 0.708–0.765, *P* < 0.001), respectively. Poor performing predictive were related to the second, four, seven and nine formulas with (AUC: 0.681, 95% CI: 0.651–0.710, *P* < 0.001), (AUC: 0.686, 95% CI: 0.656–0.716, *P* < 0.001), (AUC: 0.649, 95% CI: 0.618–0.679, *P* < 0.001), and (AUC: 0.657, 95% CI: 0.626–0.687, *P* < 0.001), respectively. Predictive value of fifth and tenth formulas were not significant (AUC: 0.507, 95% CI: 0.475–0.539, *P* = 0.751) and (AUC: 0.502, 95% CI: 0.470–0.534, *P* = 0.933), respectively. The results according to DeLong test indicated a significant difference of AUCs among the second, four, seven and nine formulas with first, third, sixth and eight formulas (*P* < 0.05). Diagnostic indices for each proposed integrated weaning indices in predicting successful weaning is presented in Table 4. Diagnostic indices in the validation dataset indicated that the first, second, third, fourth, and ninth formulae had higher accuracy which was more elaborated in continue.

**TABLE 4:** Diagnostic indices for each proposed new integrated weaning indices based on validation data set.

Formula	Cut point	SN (95% CI)	SP (95% CI)	LR + (95% CI)	LR-(95% CI)	PPV (95% CI)	NPV (95% CI)	Youden Index	Accuracy
First	145.67	92 (90–94)	45 (38–52)	1.7 (1.5–1.9)	0.18 (0.14–0.24)	87 (84–89)	59 (50–67)	0.37	82.3
Second	161.68	99 (98–100)	9 (5–14)	1.09 (1.04–1.14)	0.08 (0.03–0.21)	81 (78–83)	77 (55–92)	0.08	80.9
Third	369.44	81 (78–83)	70 (63–76)	2.7 (2.2–3.3)	0.28 (0.23–0.33)	91 (89–93)	48 (42–54)	0.51	78.5
Fourth	379.04	98 (97–99)	5 (2–9)	1.04 (1.01–1.08)	0.26 (0.11–0.60)	80 (78–83)	50 (27–73)	0.03	79.6
Fifth	333.33	74 (71–77)	24 (18–30)	0.98 (0.89–1.07)	0.9 (0.6–1.3)	79 (76–82)	19 (14–25)	0.02	63.9
Sixth	338.51	78 (75–81)	49 (42–56)	1.5 (1.3–1.8)	0.44 (0.36–0.54)	86 (83–88)	37 (31–43)	0.27	72.4
Seventh	376.12	94 (92–96)	12 (8–17)	1.07 (1.01–1.13)	0.51 (0.31–0.81)	81 (80–83)	33 (22–46)	0.06	77.3
Eighth	839.87	70 (67–74)	65 (57–71)	1.9 (1.6–2.4)	0.46 (0.40–0.54)	89 (86–91)	36 (31–41)	0.35	69.1
Ninth	93.08	95 (93–96)	13 (8–18)	1.09 (1.03–1.15)	0.42 (0.26–0.67)	81 (78–83)	38 (26–51)	0.08	78.0
Tenth	138.22	90 (88–92)	11 (6–16)	1.01 (0.96–1.07)	0.93 (0.59–1.45)	80 (77–82)	21 (14–31)	0.01	73.8

CI, Confidence interval; SN, Sensitivity; SP, Specificity; LR +, Positive Likelihood Ratio; LR-, Negative Likelihood Ratio; PPV, Positive Predictive value; NPV, Negative Predictive value.

### The cumulative effect of all new integrated weaning indices in validation dataset

Diagnostic indices in validation dataset indicated that the model by ten formulae had higher accuracy, and relatively higher values of SN, SP, LR +, PPV, and NPV and lower values of LR- in both 0.5 and optimal cutoff values as compared to two other sets of formulae. Additionally, model by second 5 formulae had higher accuracy, SN, SP, LR +, PPV, and NPV and lower values of LR- in both 0.5 and optimal cutoff values as compared to the model by first five formulae. The results indicated AUC of about, 0.83, 0.78, and 0.70 respectively, for ten, first five, and second five formulas simultaneously on predicting successful weaning after fitting a multiple logistic regression in the validation dataset (Figure 3A). The optimal cutoff values were estimated based on sensitivity and specificity trade-off in Figure 3B, showed a tradeoff of sensitivity and specificity in the cutoff of around 0.8, 0.9 and 0.8 for predicted probability in the model by ten (A), first five (B) and 2nd five (C) integrated weaning indices, respectively, in the multivariate logistic regression (Table 5).

**TABLE 5:** Diagnostic indices after multiple logistic regression model based on validation data set.

Model	SN (95% CI)	SP (95% CI)	LR + (95% CI)	LR- (95% CI)	PPV (95% CI)	NPV (95% CI)	Youden Index	Accuracy
LR on 10 formulae, cut point = 0.5	94 (93–96)	51 (44–58)	1.9 (1.7–2.2)	0.11 (0.08–0.15)	88 (86–90)	70 (62–77)	0.45	85.5
LR on 10 formulae, Optimal cut point = 0.8	85 (82–87)	72 (65–78)	3.0 (2.4–3.7)	0.21 (0.18–0.26)	92 (90–94)	54 (48–61)	0.57	82.0
LR on 1–5 formulae, cut point = 0.5	93 (91–95)	42 (35–49)	1.6 (1.4–1.8)	0.16 (0.12–0.22)	86 (84–89)	61 (52–69)	0.35	82.7
LR on 1–5 formulae, Optimal cut point = 0.9	79 (76–82)	74 (67–80)	3.1 (2.4–3.9)	0.28 (0.23–0.33)	92 (90–94)	48 (42.54)	0.53	78.3
LR on 6–10 formulae, cut point = 0.5	99 (98–100)	4 (2–8)	1.04 (1.01–1.07)	0.13 (0.04–0.42)	80 (78–83)	67 (35–90)	0.03	80.0
LR on 6–10 formulae, Optimal cut point = 0.8	73 (69–76)	65 (57–71)	2.0 (1.7–2.5)	0.43 (0.37–0.50)	89 (86–91)	38 (32–43)	0.38	70.9

LR10, Logistic regression on 10 formulae; LR 1_5, Logistic regression on first 5 formulae; LR 6_10, Logistic regression on second 5 formulae; Cut, cut point; Optimal cut point, optimal cut point based on sensitivity and specificity in the ROC curve after logistic; CI, Confidence interval; SN, Sensitivity; SP, Specificity; LR +, Positive Likelihood Ratio; LR-, Negative Likelihood Ratio; PPV, Positive Predictive value; NPV, Negative Predictive value.

## Discussion

The aim of weaning indices is to find patients who can be successfully weaned as clinical judgment is not accurate enough to predict weaning outcome in most critically ill patients (26). In this study, we introduced ten new and integrated weaning indices (index 1 to index 10). Our results showed that patients that present poor prognosis for weaning according to a high f/Vt ratio, can present better prognosis according to new integrative indices, if variables of respiratory system dynamics, the respiratory drive and the oxygenation/ventilation are appropriate. These to ten integrated indices had compared favorably to previous indices such as RSBI, NIF, and P0.1, etc. A large spectrum of weaning predictors has been studied either simple weaning indices, others that measures load and capacity e.g., negative inspiratory force (NIP), maximum inspiratory pressure (P_Imax_), tidal volume (V_T_), and breathing frequency (f) or integrative weaning indices requiring special equipment e.g., minute ventilation (V${}_{\text{E}}^{\cdot }$), the ratio of breathing frequency to tidal volume (f/VT), P0.1, and compliance, rate, oxygenation, pressure system (CROP) index (27–30). However, Conti and colleagues (28, 31), showed that vital capacity, V_T_, P0.1, V${}_{\text{E}}^{\cdot }$, respiratory frequency (RR), maximum inspiratory pressure (P_Imax_), RSBI and f/V_T_ are poor predictors of weaning outcome in an ICU population.

This fact emphasizes the hypothesis that not only the clinical evaluation, but also the evaluation of weaning indexes should be considered in the weaning process of critically ill patients. Weaning indices are based one single function/parameter have usually presented poor accuracy and for this reason, an integrative index that can evaluate multiple essential functions and may represent better outcome (32). It was reported that from 66 predictors of weaning were reviewed and analyzed by McMaster university, only eight predictors were recognized as more valuable than others (33–35), the most frequently used was the RSBI that was assessed by at least 25 studies (36). Although other variables as CROP, RSBI, P0.1, and P_Imax_ are integrative variables, but they are affected not only by the respiratory system mechanics but also could be affected by other factors such as chest and abdominal wall compliance e.g., CROP index, and by the neurological drive e.g., (P0.1) which are variable from one moment to another (37). The current indices are rather heterogeneous variables that reflect the capacity and the integrative function of the respiratory system as a whole i.e., assess both ventilator pump and also oxygenation capacity of the lungs and the ability to maintain their function and endurance effectively for a certain time interval.

To our knowledge, there are three additional integrated weaning indices (WI) reported in the literature including Huaringa et al. (38), Jabour et al. (31), and De Souza et al. (35). The first weaning index (WI1) was proposed by Huaringa et al. (38), who added two corrective factors to RSBI including the elastance index (EI = peak pressure/NIF) and the ventilator demand index (VDI = minute ventilation/10). The sensitivity of the WI1 index was 98%, specificity was 89%, PPV was 95%, NPV was 94%, and the area under the ROC curve was 0.95. Although characterizations of Huaringa’s index are excellent, further critique is required. For example, Huaringa’s study consisted of a single group with a modest number of only 59 patients. In this situation, the pilot data set did not include WI1 consisting of two added variables EI and VDI. This omission nullifies the selection of data thresholds that were derived solely from the literature. The second weaning index (WI2) was proposed by Jabour et al. (31), who combined ventilator endurance and the efficiency of gas exchange to their index. However, the interpretation of WI2 is very difficult because of the scant weaning research employing this index. Indeed, we failed at finding a single study using WI2. The current weaning indices were derived from a large patient data set with high variability of disease types aiming for the introduction of reliable, reproducible and robust weaning predictor. Nevertheless, until additional studies can validate the performance of our weaning variables, the explicit and implied limitations of our study must be taken seriously. Thus, present interpretations of our ten variables must be done with caution. Ebrahimabadi et al. (39), in their study on 105 mechanically ventilated patients showed that the integrative weaning index (IWI) as a more objective indicator has acceptable accuracy and power for predicting the 2-h SBT result. Therefore, in addition to the reliable prediction of the final weaning outcome, it has favorable power to predict if the patient is ready to breathe spontaneously as the first step to weaning which is in accordance to our results.

There were limited data in the literature regarding whether the use of IWI affected the success rate of weaning from mechanical ventilation. In our study, IWI had better predictive value for weaning patients from mechanical ventilation. Our results showed that the integration of important single functions into an index can be helpful to improve its weaning predictive value when compared with each single function component alone. Our integrative indices use essential parameters that are simple to measurement and are independent of the patient’s cooperation. The scores, in a single equation, the respiratory system dynamics, the respiratory drive, the oxygenation/ventilation, and the respiratory pattern, through NIF, P0.01, PPR-P(A-a) O_2_, SaO_2_ and RSBI ratio respectively. Several reasons concurred to the choice of the parameters above: RSBI in most papers is considered as the best or one of the best indices to evaluate the weaning outcome; respiratory system indices (Resistance and static compliance) is associated with a shorter time to weaning when compliance is more than 20 ml/cmH_2_O. Regarding oxygenation our indices use SaO_2_ and P(A-a) O_2_ which have fewer variation compared to other indices. Multiplying or dividing these indices, we can detect those patients who can or cannot maintain a good oxygenation, despite good or bad respiratory mechanics, patients who will or will not be able to maintain unassisted breathing. In that, they offer a more comprehensive perspective on pathophysiological conditions. It is proposed that the new indices maybe applied to a comprehensive continuum of hospitalized ICU patients presenting with a wide range of illnesses. Although these indices assess oxygen saturation and respiratory mechanics/drive from different views, applying each index must be adjusted according to the clinician’s goal while assessing these indices in each individual patient, i.e., the first to fifth indices are less complex than the sixth to tenth indices. Thus, the first to fifth indices are simpler and more applicable in patients with the more acceptable clinical situation while others (index six to index ten) are more suitable when confronting patients with the complex and elaborated situation since they incorporate more information reflecting the ventilator and oxygenation capacity of the respiratory pump and the lungs respectively.

Limitations of the study: Our weaning indices are more accurate than traditional and simple ones, however, it is considered that the indices aren’t completely fit in simple weaning. Furthermore, before measurement of the indices, if patients had suitable RSBI, the patients didn’t include in the measurements of new indices. Then, new indices were calculated for others (Specific populations, difficult and prolonged weaning). According to the last consensus conference, T tube or PSV 5-8 ± PEEP was recommended but in study settings, the SBT with CPAP 5 cmH2O is so common. Then, this may be an additional limitation to interpret the results. Moreover, the lack of subgroups based on ICU type and also not recorded the weaning outcome as prolong weaning can be considered as other limitations of this study. The routine clinical application of our findings should await further studies with larger samples. It should also be noted that our study population represents a heterogeneous collection and we think that it would be desirable to evaluate the validity of these indices in each one of population groups in the future. Despite these limitations, our results showed that the integration of important single functions/parameter can be helpful to improve the accuracy of successful weaning. Patients that present poor prognosis for weaning according conventional indices can present good prognosis according to the new weaning indices. Another significant point is that all previous studies were performed as comparative interventional studies whereas the current study has a cross-sectional design. It is obvious that the findings of a comparative study will be different from those of a descriptive study. According to our results, Integrative Weaning Indices compared to the physicians’ selected indexes had higher sensitivity, specificity, positive and negative predictive values, positive and negative likelihood ratios and accuracy. This was consistent with Nemer’s study in 2009 and could prove persistence of successful weaning in a 48-h period with an accuracy above 90% (37).

## Conclusion

Our ten integrated weaning indices are reliable and reproducible indices that integrate ventilator pump efficiency, pulmonary gas exchange, the balance between respiratory demands and respiratory muscle reserve into more accurate predictors of weaning success. Although the comparison of these integrated weaning indices with others weaning indices revealed better predictive power of weaning outcome in intensive care patients and can apply for a comprehensive continuum of different hospitalized patients to predict the weaning outcome, interpretations of such variables must be done with caution until further validation.

## Data availability statement

The original contributions presented in the study are included in the article/supplementary material, further inquiries can be directed to the corresponding author/s.

## Ethics statement

Ethical review and approval was not required for the study on human participants in accordance with the local legislation and institutional requirements. The patients/participants provided their written informed consent to participate in this study. The clinical trial registered code is NCT01779297 and is available at: http://clinicaltrials.gov/c.

## Author contributions

AV-A, AM, and AS designed the study. FR-B, KG-M, MK, SM, and LS contributed to acquisition of data, analysis and interpretation of data, and drafting the article. All authors contributed to the study, edited and revised manuscript, and approved final version of manuscript.

## Conflict of interest

The authors declare that the research was conducted in the absence of any commercial or financial relationships that could be construed as a potential conflict of interest.

## Publisher’s note

All claims expressed in this article are solely those of the authors and do not necessarily represent those of their affiliated organizations, or those of the publisher, the editors and the reviewers. Any product that may be evaluated in this article, or claim that may be made by its manufacturer, is not guaranteed or endorsed by the publisher.
